# Physiological responses at short distances from a parametric speaker

**DOI:** 10.1186/1880-6805-31-16

**Published:** 2012-06-13

**Authors:** Soomin Lee, Yoshihiro Shimomura, Tetsuo Katsura

**Affiliations:** 1 Center for Environment, Health and Field Sciences, Chiba University, Kashiwa, Japan; 2 Graduate School of Engineering, Chiba University, Chiba, Japan

**Keywords:** Parametric speaker, Distance, Mental task, Physiological response

## Abstract

In recent years, parametric speakers have been used in various circumstances. In our previous studies, we verified that the physiological burden of the sound of parametric speaker set at 2.6 m from the subjects was lower than that of the general speaker. However, nothing has yet been demonstrated about the effects of the sound of a parametric speaker at the shorter distance between parametric speakers the human body. Therefore, we studied this effect on physiological functions and task performance. Nine male subjects participated in this study. They completed three consecutive sessions: a 20-minute quiet period as a baseline, a 30-minute mental task period with general speakers or parametric speakers, and a 20-minute recovery period. We measured electrocardiogram (ECG) photoplethysmogram (PTG), electroencephalogram (EEG), systolic and diastolic blood pressure. Four experiments, one with a speaker condition (general speaker and parametric speaker), the other with a distance condition (0.3 m and 1.0 m), were conducted respectively at the same time of day on separate days. To examine the effects of the speaker and distance, three-way repeated measures ANOVA (speaker factor *x* distance factor *x* time factor) were conducted. In conclusion, we found that the physiological responses were not significantly different between the speaker condition and the distance condition. Meanwhile, it was shown that the physiological burdens increased with progress in time independently of speaker condition and distance condition. In summary, the effects of the parametric speaker at the 2.6 m distance were not obtained at the distance of 1 m or less.

## Background

Modern civilizations seem to be focused on achieving comfort, avoiding exposure to stresses, and removing extreme inconveniences or difficulties from living environments [1]. In the field of physiological anthropology, we should study the human nature of living in such a modern civilizations.

Recently, the parametric speaker has been used for various situations, such as an information tool in a museum and a traffic information apparatus in a station for people with visual impairments [2]. The high-directional loudspeaker systems, based on a parametric array transmit sound within a narrow range of acoustic space, like a 'spotlight'. Several studies have reported the characteristics of parametric speakers since Westervelt first explained the phenomenon of the parametric array [3]. It is well-known that the sound of a parametric speaker is heard relatively well around ±30 degrees in front of the speaker and that the sound of the parametric speaker is sharper than that of a general speaker. Ju et al. reported that the focusing of sound by a parametric speaker could be utilized to deliver audible information to people in a particular region without disturbing others [4]. In addition, with parametric speakers, it is difficult to recognize the distance of the sound source because of the lack of reverberant sound.

In our previous studies [2,5], we measured the effects of the parametric speaker sound on humans when the speakers are set at a distance of 2.6 m from the subjects. As a result, we provided the first evidence that parametric speaker sound induced less physiological stress in humans than general speaker sound, especially in relation to the cardiovascular and endocrine systems. Furthermore, we found that the reaction time to cues given by parametric speakers was shorter than that given by a general speaker.

However, nothing has yet been revealed about the effect of parametric speaker sound on physiological responses at short distances from subjects. Therefore, the purpose of this study was to clarify the physiological responses in human subjects located at a relatively short distance from a parametric speaker.

## Methods

### Subjects

Nine healthy male students participated in this study. They were asked to refrain from hard exercise, drinking caffeinated beverages, and smoking cigarettes during the 2-h period immediately preceding the experiment. The subjects performed an auditory test (ITERA, GN Otometrics, Japan) before the experiment. Their hearing ability was confirmed to be normal. All subjects gave their fully informed consent to participate in this study. Their physical characteristics are shown in Table 1.

**Table 1:** Physical characteristics of the subjects

**Subject**	**Age (yr)**	**Height (cm)**	**Weight (kg)**	**BMI**
Sub 1	25	165	65	23.9
Sub 2	23	117	62	19.8
Sub 3	22	171	57	19.5
Sub 4	23	172	69	23.3
Sub 5	24	165	47	17.3
Sub 6	24	178	71	22.4
Sub 7	23	184	70	20.7
Sub 8	24	172	54	18.3
Sub 9	22	173	65	21.7
Mean ± SD	23 ± 1.0	173 ± 6.08	62 ± 8.1	20.8 ± 2.26

BMI: Body Mass Index.

### Mental task and the sound of speaker

**Figure 1:**
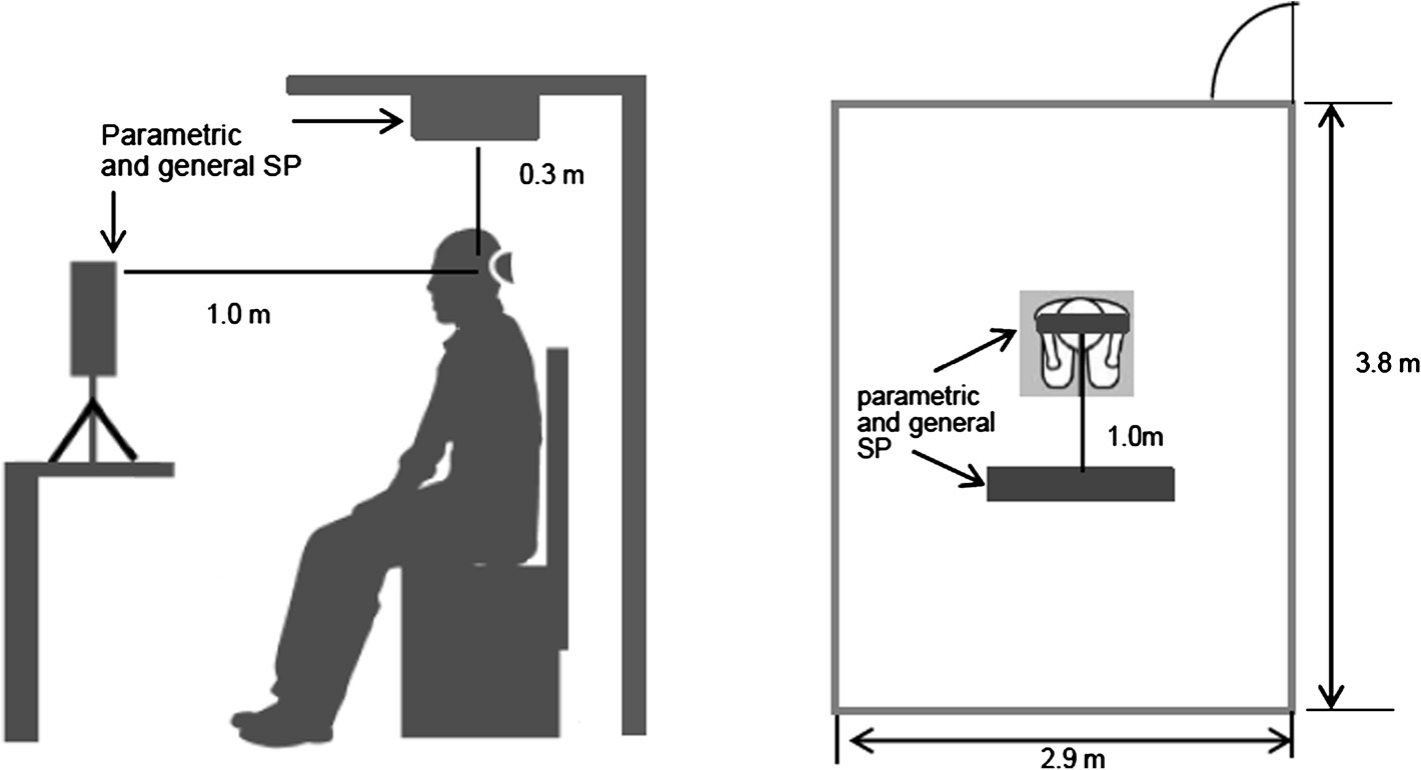
The experimental setup and layout of the soundproof room.

The experiments were conducted in a soundproof room (KF 4030, Kansai shield Co., Ltd. Japan). The size of the soundproof room is 2.9 (width) × 3.8 (depth) × 2.3 (height) m. The effect of the sound proofing is 25 dB at 1 kHz (Figure 1). The mental task consisted of both normal and deviant sentences coming from the speakers. In the experiment, the speakers were consisted of a general speaker system (W2-800SL, Tangbamd, Japan) and a parametric speaker system (ultrasonic wave 40 kHz with 19 actuators). In addition, a frequency analyzer (SpectraLab, Sound Technology, Germany), an amplifier (RDA-212, RASTEM Systems) and a noise meter (LA-1440, ONOSOKKI, Japan) were used to analyze the sound. The sound of the parametric speaker was made by the following methods. Airborne ultrasound waves of 40 kHz were dynamically double-sideband-AM modulated with audio signals, after being radiated from ultrasound emitters. The inherent nonlinearity of the air works as a de-modulator. Thus, de-modulated sounds having sharp beamwidths impinge on the ear drums.

**Figure 2:**
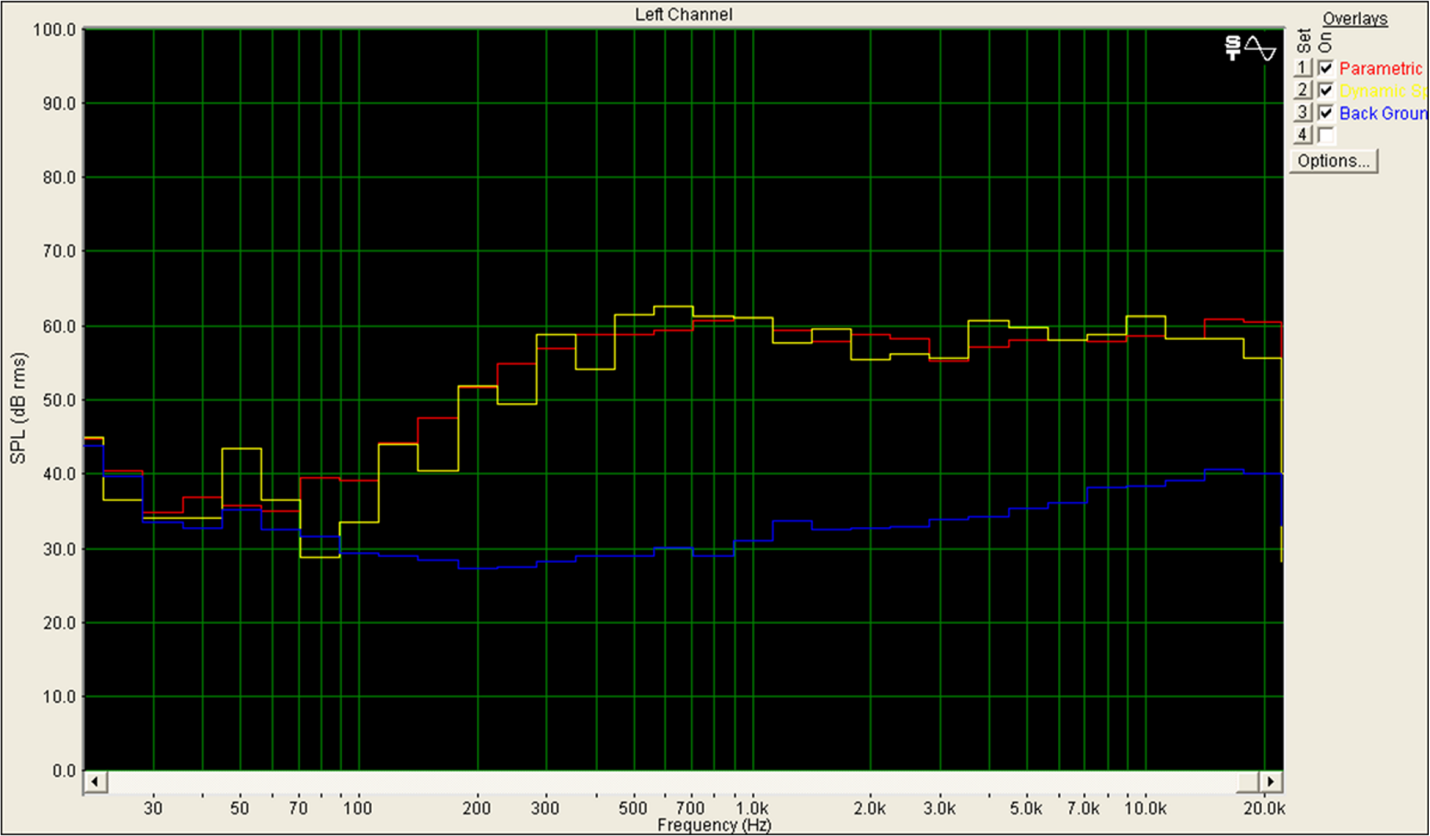
**The output characteristic of the general speaker and parametric speaker.** Red, parametric speaker; Yellow, general speaker; Blue, background of the soundproof room.

Subjects were instructed to judge whether the sentence was correct or incorrect and accordingly, to push one of two buttons as quickly as possible. The sentences were made by a text-to-speech synthesis software (SMART TALK Version 3, OKI, Japan). For example, a normal (“correct”) sentence is “shounen ga ie ni kaeru.” (a boy comes back home). In this case, the subjects were asked to push a red (correct) button. By contrast, a deviant (“incorrect”) sentence is “shounen ga ie ni taberu” (a boy eats home). In this case, the subjects were asked to push a blue (incorrect) button. The LAeq of the sound generated by the general speaker was 72.4 dBA, and the LAeq of sound generated by the parametric speaker was 72.3 dBA. The background LAeq of the soundproof room was 52.2 dBA. The LAeq was measured using an artificial head measurement system (HMS IV-AACHENHEAD, Headacoustics, Germany) during 10 s. The measurement position of the LAeq was at the ear of the artificial head measurement system. The adjustment of the frequency characteristic was conducted using DSP Board (ADAU1401, Analog devices, U.S).The Figure 2 shows the output characteristic of the general speaker and parametric speaker.

### Protocol

Four experiments were conducted at the same time of day on separate days and under the same conditions with the exception of the speaker condition (general speaker or parametric speaker) and distance condition (0.3 m or 1.0 m). After the subjects entered the soundproof room, they were asked to relax for at least 15 minutes before the recording sessions began. Subjects completed three consecutive sessions: a 20-minute quiet period as a baseline, a 30-minute mental task period with a general speaker or parametric speaker, and a 20-minute recovery period. In the previous study [2], there was not the physiological change in the subjects 30 minuters after the mental task. Therefore, we set 30 minutes as the lenght of the mental task in this experiment. Subjects were told to rest and physically relax throughout the experimental period. The order of the two speaker conditions and two distance conditions were counterbalanced between the subjects.

### Physiological parameters

EEG activity was recorded with Ag/AgCl electrodes affixed with electrode paste on the Pz, Cz, Fz, O1, and O2 electrode sites of the international 10-20 system. Linked earlobes were used as a reference with a forehead ground. A bipolar electrooculogram (EOG) was recorded with electrodes placed above and below the left eye. The EEG and EOG were amplified by appropriate devices (EEG100B and EOG100B, BIOPAC Systems, U.S). EEGs were fast Fourier transformed for each 5.12 s of data, not including artifacts such as ocular movement. We then obtained the relative power density of the alpha wave (8 13 Hz/4 to 30 Hz) [6].

An electrocardiogram (ECG) was recorded using an amplifier (ECG 100B, BIOPAC Systems), and digitized to derive heart rate variability (HRV) and heart rate (HR) using signal processing software (Mathcad, PTC, Japan). The high-frequency (HF) component and low-frequency (LF) component were integrated at 0.05 to 0.15 Hz and 0.15 to 0.40 Hz of the power spectra, respectively [7,8]. Sympathetic nervous activity (LF/HF) and parasympathetic nervous activity (HF/(LF + HF)) were calculated. A photoplethysmogram (PTG) was recorded using an amplifier (PPG 100B, BIOPAC Systems), a low-pass filter was set at 3.0 Hz, and a high-pass filter was set at 0.5 Hz. We measured the b/a ratio of the second derivative of PTG (SDPTG), which was considered to reflect the distensibility of the vascular wall [9,10].

All signals of the physiological indexes were converted from analog to digital at a 1 kHz sampling rate (MP150, BIOPAC Systems) and were stored in a computer. Continuous measurements of beat-to-beat BP were obtained with an ambulatory noninvasive BP monitor (TNO, Portapres, Netherlands).

### Statistical analyses

We calculated the mean of each five-minute period for all indexes. In the physiological parameters, changes (Δ) calculated by subtracting the respective baseline values from the average values for the task period and recovery period were used to conduct statistical analyses. For the physiological responses, a three-way repeated-measures ANOVA (speaker factor × distance factor × time factor) was conducted. By contrast, in the task performance, a two-way repeated-measures ANOVA (speaker factor × distance factor) was conducted. All statistical analyses were performed using SPSS 11.0 J (SPSS, U.S). Differences with values of *p* < 0.05 were considered significant. Data are shown as means ± standard errors unless otherwise stated.

## Results

### Physiological data

Table 2 showed that the HR, HRV, BP and the relative power density of the alpha wave at Fz, Cz and O1 were not significantly different between the two speaker conditions and the two short distance conditions. However, most of physiological responses showed that the main effects of the time factor were significant.

**Table 2:** Changes of Δsympathetic, Δparasympathetic, ΔHR, ΔSBP, ΔDBP and ΔEEG alpha-band ratio

			**speaker condition**		**distance condition**		**time condition**	
			**general**	**parametric**	**0.3m**	**1.0m**	**task period**	**recovery period**
Δ** Sympathetic nerve activity**			1.152 ± 0.161	1.326 ± 0.174	1.004 ± 0.150	1.047 ± 0.182	1.162 ± 0.306 *	1.796 ± 0.351
Δ**Parasympathetic nerve activity**			-0.079 ± 0.011	-0.102 ± 0.013	-0.090 ± 0.013	-0.091 ± 0.012	-0.089 ± 0.024 +	-0.113 ± 0.022
Δ**Heart rate**			-1.047 ± 0.318	-0.770 ± 0.284	-0.454 ± 0.276	-1.363 ± 0.321	-0.719 ± 0.492 **	2.170 ± 0.726 *
Δ**SBP**			0.389 ± 0.700	-0.602 ± 0.631	1.028 ± 0.673	-1.241 ± 0.645	-0.702 ± 1.226 +	0.500 ± 1.096
Δ**DBP**			6.176 ± 0.774	6.343 ± 0.971	5.148 ± 0.884	7.370 ± 0.859	6.597 ± 1.397 ***	10.500 ± 0.719 **
Δ**α brand ratio at Fz**			-0.008 ± 0.003	-0.002 ± 0.003	-0.003 ± 0.003	-0.007 ± 0.003	0.001 ± 0.006	0.014 ± 0.007
Δ**α brand ratio at Cz**			-0.003 ± 0.003	0.006 ± 0.003	-0.002 ± 0.003	0.003 ± 0.003	0.010 ± 0.006	0.008 ± 0.005
Δ**α brand ratio O1**			0.007 ± 0.002	0.015 ± 0.004	0.009 ± 0.003	0.014 ± 0.003	0.018 ± 0.007	0.007 ± 0.004

+: *P* <0.1, *: *P* <0.05, **: *P* <0.01, ***: *P* <0.001.

**Figure 3:**
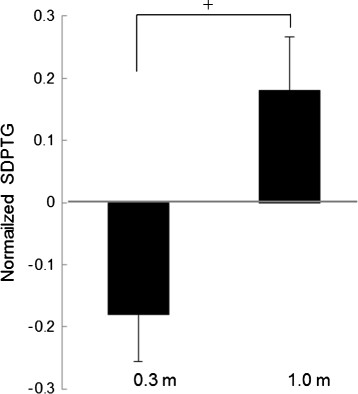
**Normalized SDPTG in the 0.3 m and 1.0 m conditions.** (means ± S.E., +: *p* < 0.1).

**Figure 4:**
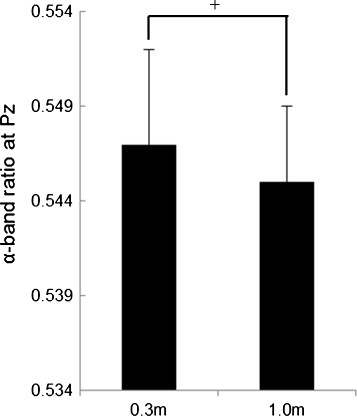
**Relative power density of the alpha wave at Pz in the 0.3 m and 1.0 m conditions.** (means ± S.E., +: *p* < 0.1).

**Figure 5:**
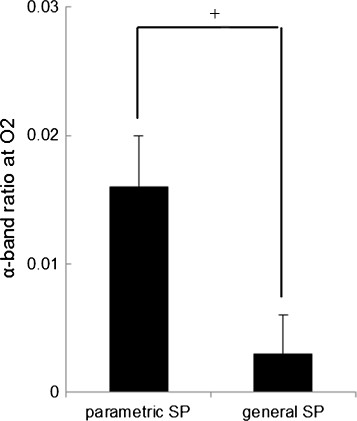
**ΔRelative power density of the alpha wave at O2 in the parametric and general speaker condition.** (means ± S.E., +: *p* < 0.1).

Figure 3 shows the results of the normalized SDPTG (Z-score). The normalized SDPTG in the 1.0 m condition tended to be significantly higher than that in the 0.3 m [F(1,8) = 4.908, *p* = 0.0576] condition. Figure 4 shows the results of the relative power density of the alpha wave at Pz. The relative power density of the alpha wave at Pz in the 1.0 m condition tended to be significantly higher than that in the 0.3 m condition [F(1,8) = 3.662, *p* = 0.0920]. Figure 5 shows the results of the Δ in relative power density of the alpha wave at O2, for which the parametric condition tended to have a significantly higher value than the general condition [F (1, 8) = 4.428, *p* = 0.0685]. The repeated measures ANOVA revealed no significant interactions of the three factors in all physiological data.

### Task performance

**Figure 6:**
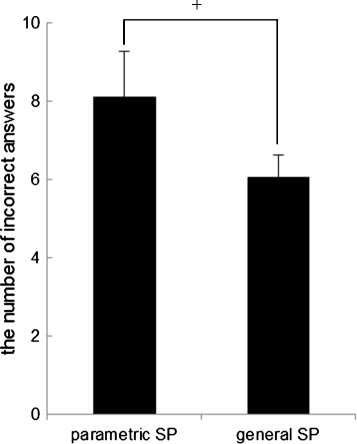
**The number of incorrect answers in the parametric speaker condition and general speaker condition.** (means ± S.E., +:*p* < 0.1).

The reaction time was not significantly different between speaker condition and distance condition for both normal sentences and deviant sentences. However, Figure 6 shows that the number of incorrect answers for the parametric condition tended to be significantly higher than that for the general speaker (F (1, 8) = 4.850, *p* = 0.0588). It was shown that there were no significant differences between the 0.3 m and 1.0 m conditions for both normal sentences and deviant sentences. The repeated measures ANOVA revealed no significant interactions of the two factors in all task performance data.

## Discussion

Recently, a new type of speaker called the parametric speaker has been developed to generate highly directional sound, and it is now commercially available. In our previous studies, we demonstrated that the sound of a parametric speaker set at a distance of 2.6 m from the subjects was less stressful than that of the general speaker with regard to the cardiovascular system and endocrine system [2,5]. However, nothing has yet been demonstrated about the effect of the sound of parametric speakers at shorter distances from subjects. Therefore, in the present study we measured the physiological responses to the sound by a general speaker and a parametric speaker at short distance conditions (0.3 m and 1.0 m).

Table 3 combines the results of the present study with those of our previous study [2], in which we verified the effects of the parametric speaker sound on physiological functions at the 2.6 m distance. In particular, we elucidated that the Δ in sympathetic nervous activity in the parametric condition was significantly lower than that in the general condition (F(1,8) = 10.024, *p* < 0.05). Furthermore, we showed that the Δ in parasympathetic nervous activity in the parametric condition tended to be significantly smaller than that in the general condition (F(1,8) = 6.092, *p* < 0.1). ΔSDPTG for the parametric speaker tended to be significantly higher than that for the general speaker (F(1,8) = 4.245, *p* < 0.1). We considered that the characteristics of the parametric speaker had an influence on articulation and reverberation of the sound. There has been no direct report on articulation and reverberation of parametric speaker sound. However, it has been reported that the sound focusing of a parametric speaker could be utilized to deliver audible information to people in a particular region without disturbing others [4] because the directivity was so strong. Furthermore, Nabelek et al. verified that the articulation of sounds was decreased by reverberation [11]. We supposed that a parametric speaker has less reverberation and higher articulation because of its strong directivity.

**Table 3:** The comparisons of the distance between general speaker and parametric speaker

	**0.3 m**		**1.0 m**		**2.6 m**	
	**parametric SP**	**general SP**	**parametric SP**	**general SP**	**parametric SP**	**general SP**
∆Sympathetic nerve activity	1.279 ± 0.301	0.631 ± 0.240	1.215 ± 0.338	1.551 ± 0.345	0.527 ± 0.559	1.616 ±0. 484*
∆Parasympathetic nerve activity	-0.108 ± 0.028	-0.072 ± 0.024	-0.087 ± 0.025	-0.087 ± 0.020	-0.037 ± 0.005	-0.152 ± 0.045 +
∆SDPTG	-0.056 ± 0.018	0.027 ± 0.012	0.055 ± 0.013	0.064 ± 0.026	0.834 ± 0.033	0.802 ± 0.026 +
∆α band ratio at Pz	-0.001 ± 0.009	0.002 ± 0.003	0.015 ± 0.004	0.002 ± 0.004	0.040 ± 0.024	0.034 ± 0.083
reaction time	0.496 ± 0.097	0.876 ± 0.082	0.887 ± 0.089	0.939 ± 0.109	0.778 ± 0.044	0.852 ± 0.044 *

#2.6 m, results of our previous study (Lee *et al*., 2011).

*, parametric SP *vs* general SP (*P* <0.05).

+, parametric SP *vs* general SP (*P* <0.1).

We hypothesized that the better audibility of the parametric speaker sound might result in fewer physiological burdens. From this perspective, we verified in our previous study that the sound of a parametric speaker that was set at a 2.6-m distance from subjects was indeed less stressful than that of the general speaker with regard to the cardiovascular system [2].

Meanwhile, in the present study, most physiological responses only showed the significant main effects of the time factor, which suggested that the physiological burden increased by processing time at distances of 1 m or less. No physiological measurement items were significantly different between the speaker conditions.

When speakers are set a good distance from subjects, we can presume that a general speaker has more reverberation and lower articulation compared to a parametric speaker. However, we estimated that there was little difference in articulation between the parametric and general speaker sounds at the shorter distance. At the shorter distance, it is possible that the subjects perceived that the general speaker sound was nearly equal to the parametric speaker sound because the reverberation of the conventional speaker decreases.

Therefore, we considered that there is little effect of the directional characteristic of the parametric speaker at distances of 1 m or less. We expected that there was little difference in the articulation between speaker conditions because reverberations from wall surfaces might be hard to detect at such short distances (1 m or less). As a result, the speaker condition might not cause any physiological difference. In this study, we verified that the subjects felt no difference between general speaker sound and parametric speaker sound at distances of 1 m or less. In other words, these results suggest that a farther speaker distance might be necessary to exploit the beneficial characteristics of the parametric speaker and be better for the human body since, at a distance of 2.6 m, the physiological burden of the parametric speaker sound is lower than that of the general speaker. These results were obtained from the viewpoint of physiological anthropology, which will be important for future studies to consider regarding the applications of parametric speaker sound.

## Abbreviations

ECG, electrocardiogram; EEG, electroencephalogram; EOG, electrooculogram.

## Competing interests

The authors have no competing interests to declare.

## Authors’ contributions

SL performed the experiments and wrote the manuscript, analyzed the data. TK conceived and designed the study. SL, YS and TK were responsible for coordination of the study and oversight of data collection and analysis. All authors have read and approved the final manuscript.
